# Pregnancy-Associated Plasma Protein-A2 Is Associated With Mortality in Patients With Lung Cancer

**DOI:** 10.3389/fendo.2020.00614

**Published:** 2020-09-02

**Authors:** Rikke Hjortebjerg, Ulrick Espelund, Torben Riis Rasmussen, Birgitte Folkersen, Torben Steiniche, Jeanette Bæhr Georgsen, Claus Oxvig, Jan Frystyk

**Affiliations:** ^1^Medical Research Laboratory, Department of Clinical Medicine, Aarhus University, Aarhus, Denmark; ^2^Department of Molecular Endocrinology (KMEB), University of Southern Denmark, Odense, Denmark; ^3^Steno Diabetes Center Odense, Odense University Hospital, Odense, Denmark; ^4^Department of Endocrinology and Internal Medicine, Aarhus University Hospital, Aarhus, Denmark; ^5^Department of Pulmonary Medicine, Aarhus University Hospital, Aarhus, Denmark; ^6^Department of Pathology, Aarhus University Hospital, Aarhus, Denmark; ^7^Department of Molecular Biology and Genetics, Aarhus University, Aarhus, Denmark; ^8^Department of Endocrinology, Odense University Hospital, Odense, Denmark

**Keywords:** insulin-like growth factor, insulin-like growth factor binding protein, lung cancer, mortality, pregnancy-associated plasma protein-A, pregnancy-associated plasma protein-A2

## Abstract

Pregnancy-associated plasma protein-A (PAPP-A) and its homolog PAPP-A2 are enzymes that modulate the availability and mitogenic activity of insulin-like growth factor-I (IGF-I). PAPP-A has been implicated in numerous cancers but reports on PAPP-A2 in malignancy are non-existent. In a prospective observational study of 689 patients under suspicion of lung cancer, we examined levels of PAPP-A and PAPP-A2 and their relationship with mortality. Serum PAPP-A and PAPP-A2 concentrations were determined in pre-diagnostic blood samples using ELISA, and immunohistochemical staining of PAPP-A and PAPP-A2 was performed in malignant tissue from five operable patients. A total of 144 patients were diagnosed with lung cancer, whereas the diagnosis was rejected in 545 subjects, who served as a control group. PAPP-A2 concentrations were higher in patients with lung cancer [median (IQR): 0.33 (0.21–0.56) ng/mL] than in controls [0.27 (0.17–0.39) ng/mL], *p* < 0.001, whereas PAPP-A levels did not differ. Presence of PAPP-A and PAPP-A2 were confirmed in tumor specimens, and staining occurred in a heterogeneous pattern. Patients were observed for a median (range) of 7 (6; 8) years, during which 114 patients (79.2%) died. Patient mortality differed according to PAPP-A2 tertile (*p* < 0.001). PAPP-A2 was associated with mortality with an unadjusted hazard ratio (95% CI) per doubling in protein concentration of 1.30 (1.12; 1.53), *p* = 0.001. In a multivariable model adjusted for age, sex, and BMI, PAPP-A2 remained predictive of the endpoint with a hazard ratio per doubling in protein concentration of 1.25 (1.05; 1.48), *p* = 0.013. Collectively, PAPP-A2, but not PAPP-A, is elevated in patients with lung cancer and associated with mortality. This novel role of PAPP-A2 in cancer warrants further functional studies as well as validation in external cohorts.

## Introduction

Lung cancer is one of the most common human malignancies worldwide with considerable attendant societal costs. Tumor heterogeneity and the lack of seromarkers for detection of the disease at early stages pose a formidable challenge and contribute to high mortality rates. Insulin-like growth factor I (IGF-I) is a pivotal player in the multifaceted process of malignant disease, including lung cancer, and signaling through the IGF-I receptor (IGF-IR) stimulates mitogenesis, metabolism, and anti-apoptosis (1, 2).

Pregnancy-associated plasma protein-A (PAPP-A) and PAPP-A2 comprise the only two known members of the pappalysin family of metalloproteinases, sharing 45% amino acid identity (3, 4). They are responsible for proteolytic cleavage of a subset of IGF-binding proteins (IGFBPs), through which they increase IGF availability and potentiate its growth stimulatory effects (5). PAPP-A has been suggested as an accomplice in several types of cancer (6–9) and has been extensively studied due to its biomarker potential (3, 10–13). Although PAPP-A2 was recently established as a regulator of the IGF axis in human physiology (14), the biology of PAPP-A2 is poorly understood compared to PAPP-A (15), and there are currently no reports linking PAPP-A2 protein and cancer mortality (9).

PAPP-A specifically cleaves IGFBP-2,−4, and−5 and is widely expressed in multiple tissues, including those of tumor origin, where it tethers to cell surfaces (16, 17). Thus, PAPP-A causes a release of bioactive IGF in close proximity to the IGF-IR. Shifts in PAPP-A levels have been suggested to modify the relationship between bound and free IGF in various neoplasms (8, 18–20). In patients with lung cancer, serum PAPP-A levels have been shown to be elevated (19), and down-regulation of PAPP-A expression decreases lung cancer progression *in vivo* (21). The present authors previously described a cohort of women with ovarian cancer, in which PAPP-A levels were investigated in serum and malignant ascites (20). In ascites, which surrounds the ovarian tumor in the abdominal cavity and is a negative prognostic factor, PAPP-A levels were 46-fold higher as compared to serum from the same patient. It was further shown that the ability of ascites to activate the IGF-IR *in vitro* was increased by 31% as compared to serum, and immunohistochemistry (IHC) of ovarian tumor specimens revealed abundant staining of both IGF-IR and PAPP-A.

Similar to PAPP-A, placentally derived PAPP-A2 is abundantly present in the circulation throughout pregnancy, but the protein is also detectable in non-pregnant men and women (22). However, PAPP-A2 has generally not been investigated in human pathologic conditions outside pregnancy. PAPP-A2 exhibits proteolytic activity against IGFBP-3 and−5, but unlike PAPP-A, PAPP-A2 does not show surface tethering (15). Recently, Dauber et al. (14) reported the first human PAPP-A2 deficiency cases, who presented with short stature and severe perturbations in the IGF system. This finding provided conclusive evidence of the importance of PAPP-A2 in human physiology.

The present study evaluated PAPP-A and PAPP-A2 levels in serum from 689 patients under suspicion of lung cancer and assessed PAPP-A and PAPP-A2 expression by IHC in surgical specimens. Furthermore, we investigated the associations of PAPP-A and PAPP-A2 with mortality in the 144 patients with a cancer diagnosis and compared their prognostic performances.

## Methods

### Patient Characteristics

The Department of Pulmonary Medicine at Aarhus University Hospital receives patients under suspicion of lung cancer referred from their general practitioner or other hospital departments within the region of Aarhus, Denmark. All referred patients are examined in a fast-track diagnostic setup, where medical examination, routine biochemistry, CT, PET, lung function tests, endosonography, and biopsies are performed within four weeks of their first visit.

**Figure 1:**
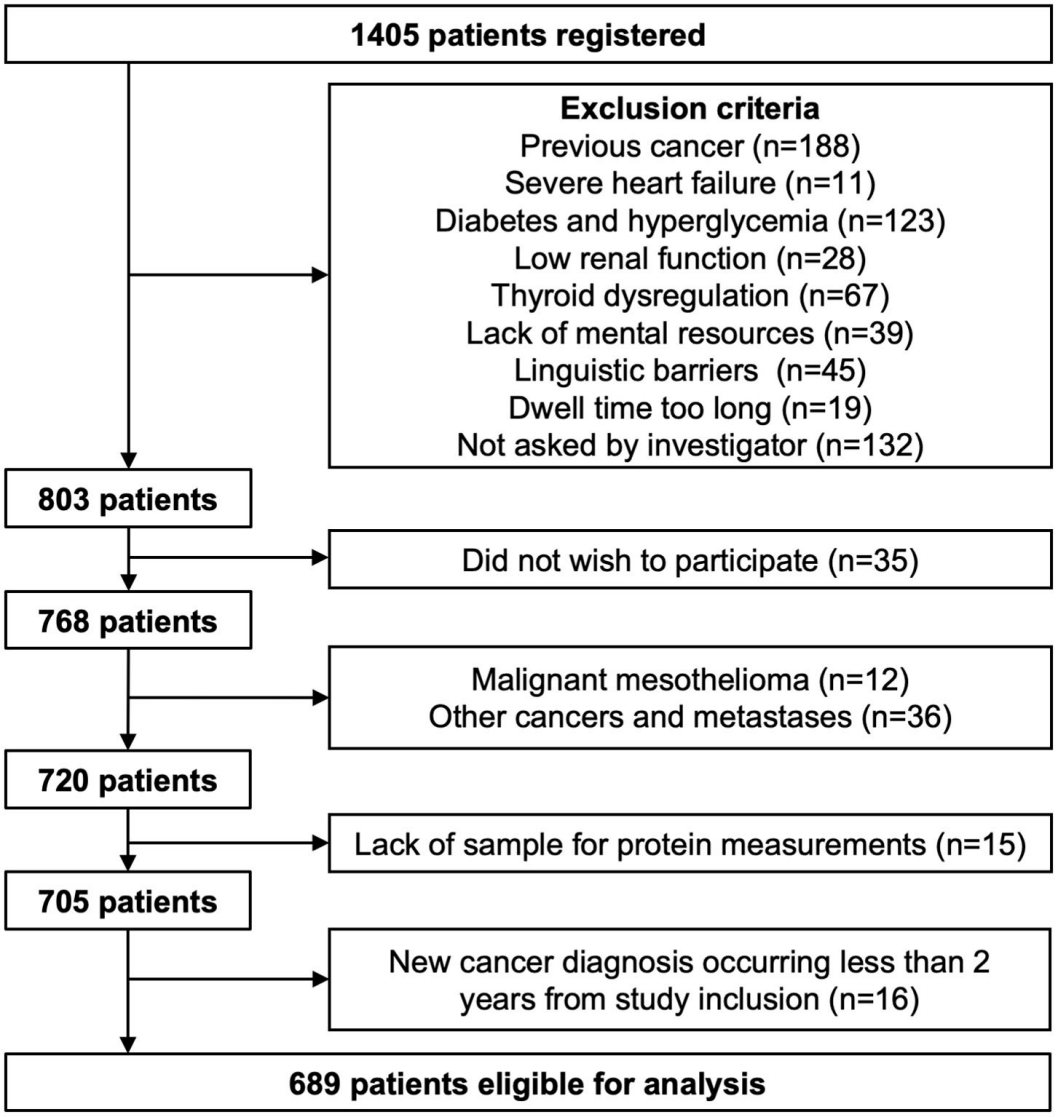
Flow chart of patient inclusion. Some patients met more than one exclusion criteria; hence the sum does not equal the reduction in patient number.

All patients referred from February 2009 through April 2011 were invited to participate in the present study at their first visit. A total of 1,405 patients were registered, and information was obtained on smoking habits, symptoms including dyspnea, height, weight, recent weight gain or loss, and reasons for exclusion when applicable. These data were paired with routine biochemistry, diagnoses given at the end of the diagnostic course, lung function tests, diagnoses given in conjunction with previous contacts with the Danish health system, and Charlson comorbidity index. The TNM system was used to stage the cancer; T describing the size of the primary lung tumor, N describing regional lymph node involvement and M describing distant metastasis. These values were combined to assign an overall cancer stage (1–4). Project blood samples were collected in conjunction with the routine samples, centrifuged, separated in aliquots, and stored at −80° until assay. Exclusion criteria were previous malignancies apart from non-melanoma skin cancer (*n* = 188), severe heart failure (NYHA III/IV) (*n* = 11), thyroid dysregulation (*n* = 67), lack of mental resources (23), linguistic and cultural barriers (*n* = 45) and long dwell time (*n* = 19). Of all registered cases in the period, 132 patients were not asked by the investigator. In addition, biochemical criteria were applied to identify patients with poorly managed diabetes and decreased renal function. Patients with diabetes and an HbA1c above 7.0% DCCT were excluded from the study (calculated as an average of all available measurements across the study period) (*n* = 123). This level equals 53 mmol/mol by the IFCC standard and reflects an estimated average plasma glucose of 8.5 mmol/l. Patients with an estimated glomerular filtration rate (eGFR) below 40 ml/min were excluded (determined by the MDRD formula without correction for ethnicity) (*n* = 28). Exclusion criteria reduced the cohort to 803 patients. Of these, 35 did not wish to participate. Some patients were subsequently diagnosed with malignant mesothelioma (*n* = 12) and other cancers than lung cancer including metastases (*n*=36), and these two groups were excluded from analyses. Finally, patients were also excluded due to lack of sufficient blood for determination of target proteins (*n* = 15), resulting in a total of 705 patients available for laboratory measurements. However, to ensure that all variables were investigated in the context of lung cancer, patients were excluded if a new cancer diagnosis occurred <2 years from study inclusion (*n* = 16). Patients that received a new cancer diagnosis more than 2 years into follow-up were included in the study (*n* = 34). A total of 689 patients were eligible for analysis (Figure 1). Hereof, 144 patients (20.9%) were diagnosed with lung cancer, whereas this diagnosis was rejected in 545 patients. Patients were grouped as follows: controls (control, *n* = 545), small cell lung carcinoma (SCLC, *n* = 13), non-small cell lung carcinoma, NSCLC, adenocarcinoma subtype (NS-Ad, *n* = 75), NSCLC, squamous cell subtype (NS-Sq, *n* = 27), and NSCLC, other subtypes than NS-Ad and NS-Sq (NS-x, *n* = 29).

Baseline descriptions of controls and patients are summarized in Table 1, including the clinical stage in lung cancer patients. Patients were allocated to treatment independent of study participation. A total of 117 controls were diagnosed with pneumonia, hemoptysis, sarcoidosis, or other respiratory diseases. Control subjects where a malignant cause was ruled out were given an unspecific “observation” diagnosis (*n* = 303), whereas others showed an abnormal computerized tomography scan (*n* = 182). Written informed consent was obtained from all patients, and the study (id: 1-10-72-155-12) was approved by the Central Denmark Region Committees on Biomedical Research Ethics (IRB 0005129). The study was conducted in accordance with the Declaration of Helsinki.

**Table 1:** Baseline and survival characteristics.

**Characteristics**	**Control**	**Cancer**	**Cancer subtype**			
			**SCLC**	**NS-Ad**	**NS-Sq**	**NS-x**
Number, *n*	545	144	13	75	27	29
Males, *n* (%)	266 (48.8)	66 (45.8)	6 (42.2)	23 (30.7)^**^	18 (66.7)	19 (65.5)
Age, years	61.9 ± 13.1	67.1 ± 10.6^**^	67.7 ± 10.9	64.7 ± 10.0	72.8 ± 9.6^**^	67.5 ± 11.4^*^
BMI, kg/m^2^	25.5 ± 4.9	23.6 ± 3.7^**^	25.6 ± 3.6	23.1 ± 3.5^**^	23.9 ± 3.6	23.5 ± 4.4^*^
CRP (mg/L)	2.6 (0.9; 6.8)	13.9 (3.6; 32.9)^**^	5.8 (2.4; 22.8)^*^	8.7 (3.1; 24.0)^**^	32.5 (20.6; 48.7)^**^	13.1 (4.4; 33.9)^**^
eGFR	85 (74; 99)	90 (79; 106)^**^	86 (81; 101)	88 (78; 103)	91 (79; 112)	93 (83; 108)
**Smoking status**, ***n*** **(%)**						
Never	117 (21.5)	7 (4.6)	0 (0.0)	4 (5.3)	1 (3.7)	2 (6.9)
Former/current	368 (67.5)	131 (91.0)	13 (100.0)	69 (92.0)	25 (92.6)	24 (82.8)
Unknown/missing	60 (11.0)	6 (4.2)	0 (0.0)	2 (2.7)	1 (3.7)	3 (10.3)
**Stage**, ***n*** **(%)**						
1		27 (18.8)	2 (15.4)	17 (22.7)	2 (4.4)	6 (20.7)
2		14 (9.7)	0 (0.0)	7 (9.3)	4 (14.8)	3 (10.3)
3		29 (20.1)	5 (38.5)	6 (8.0)	13 (48.2)	5 (17.2)
4		56 (38.9)	4 (30.8)	35 (46.7)	5 (18.5)	12 (41.4)
Unknown		18 (12.5)	2 (15.4)	10 (13.3)	3 (11.1)	3 (10.3)
PAPP-A, ng/mL	1.03 (0.84; 1.27)	1.04 (0.86; 1.36)	1.02 (0.90; 1.54)	1.01 (0.84; 1.26)	1.10 (0.78; 1.43)	1.02 (0.90; 1.26)
PAPP-A2, ng/mL	0.27 (0.17; 0.39)	0.33 (0.21; 0.56)^**^	0.33 (0.22; 0.78)	0.29 (0.20; 0.47)	0.47 (0.24; 0.67)^**^	0.34 (0.23; 0.49)^*^
Survival, days		377 (190; 1,301)	550 (324; 1,717)	397 (206; 1,535)	281 (141; 899)	307 (157; 865)
Mortality at endpoint, *n* (%)	78 (14.3)	114 (79.2)	10 (76.9)	59 (78.7)	23 (85.2)	22 (75.9)

*Baseline and survival characteristics in controls, patients with lung cancer and subtypes of lung cancer. Patients are grouped as follows; control subjects (Control), all cancer patients, (Cancer), small cell lung carcinoma (SCLC), non-small cell lung carcinoma (NSCLC), NSCLC adenocarcinoma subtype (NS-Ad), NSCLC squamous cell subtype (NS-Sq), and NSCLC other subtypes than NS-Ad and NS-Sq (NS-x). Survival refers to median survival time and comprises all cancer patients who were censored or experienced an event. Categorical variables are indicated as number (n) and percentage (%) of patients, and continuous variables are mean ± SD or median (25th percentile; 75th percentile)*.

* *p < 0.05*,

** *p < 0.005 as compared to controls*.

*BMI, body mass index; CRP, C-reactive protein; eGFR, estimated glomerular filtration rate; PAPP-A, pregnancy-associated plasma protein-A*.

### Outcome Measures

All-cause mortality for lung cancer patients was recorded until March 2017. The 144 patients with a cancer diagnosis had a median (range) survival of 377 days (14 days−8 years). Since the first cancer patient was included in March 2009 and the final patient in March 2011, all cancer patients were followed for at least 6 years [median (range): 7 (6; 8) years]. Survival was documented for each individual using the Danish Civil Registration System and the National Causes of Death Registry, which offers information from physicians on causes of death according to the International Classification of Diseases, Tenth Revision (ICD-10). Because of the high-quality Danish registration system, no patients were lost during follow-up.

### Laboratory Measurements

Routine biochemistry was performed at the hospital's laboratory using widely available automated assays. Serum protein levels of PAPP-A and PAPP-A2 were measured using PAPP-A (AL-101) and PAPP-A2 (AL-109) ELISA kits (AnshLabs, Webster, TX, USA). All samples were analyzed in a blinded fashion in random order.

### Immunohistochemistry

A subgroup of five patients who were operable provided tumor tissue for IHC, which was performed as previously described (20) using antibodies specific for PAPP-A (PAC1-D8-mIgG2a) (24) and PAPP-A2 (P257) (22) at 10 and 20 mg/L, respectively.

### Statistical Analysis

The assumption of normality was checked using quantile-quantile plots and by the Shapiro-Wilk test, and non-normally distributed variables were transformed prior to statistical analyses. Whenever possible, parametric statistical tests were applied. Groups were compared with Student's *t*-test (two groups) or one-way ANOVA and *post-hoc* tests with Bonferroni's correction (multiple groups). If there was evidence against the assumption of equal variance by Bartlett's test, or if data did not follow a normal distribution, Wilcoxon rank-sum test or Kruskal-Wallis test was applied, respectively. Categorical variables were evaluated by χ^2^-test. PAPP-A and PAPP-A2 were modeled categorically as tertiles and as continuous variables after log transformation using log(protein)/log(2). Accordingly, one unit increase in protein level on the log_2_-scale corresponds to a doubling in protein. Test for linear trend (continuous protein level) across ordered groups (cancer stage) was performed by linear regression analyses with the ordered group as a continuous explanatory variable with equal distance between steps. Test for ordered categorical trend (protein tertile) across ordered groups was performed using an extension of the Wilcoxon rank-sum test developed by Cuzick (25).

The area under the receiver operating characteristic (ROC) curve (AUC) was used to assess the prognostic ability of PAPP-A and PAPP-A2. However, AUC is a metric for binary classification and does not consider individual survival times and censoring. As proposed by Harrell et al. (26) as an extension of AUC, the concordance (C) index was used as a measure of concordance between the protein of interest and the possibly censored survival outcome, using a similar range from 0 to 1. Suggested by Pencina et al. (27, 28), the Harrell's C index is the most appropriate in capturing the discriminating ability of a prognostic variable to separate subjects with varying survival time and outcome status.

Kaplan-Meier survival curves were performed for PAPP-A and PAPP-A2 tertiles, and incidence distributions were compared using the log-rank test. Cox proportional hazards models were developed to explore associations between survival endpoint and the explanatory variable, using both the continuous variable and tertiles with the low tertile as reference group. Hazard ratios (HRs) assessed the risk of death in unadjusted models and after adjustment for *a priori* defined covariables; age, sex, and BMI. Smoking status was not associated with PAPP-A or PAPP-A2 level, and hence, not included. The validity of the proportional hazards and linearity assumptions were checked by log-log plots, fitted survival curves and smoothed martingale and Schonenfeld residuals plot; no deviations from proportionality were identified (26, 29, 30).

Results are presented as mean ± SD for normally distributed data and median (25th percentile; 75th percentile) for skewed data. AUC, C-statistics, and HRs are presented with 95% confidence intervals (CI). Two-tailed *P-*values < 0.05 were considered statistically significant. Data were analyzed using Stata software (StataCorp LP, College Station, TX, USA). Harrell's C index and Somers' D statistics for censored data was calculated using the “somersd” module in Stata version 13.

## Results

### Baseline Characteristics

Control and patient characteristics by subtype of lung cancer are given in Table 1. Cancer patients were significantly older than controls and had a lower BMI, and a higher percentage were current or former smokers. Among cancer subtypes, patients were similar with regards to age and BMI, and disease stage did not differ between cancer subgroups. However, as expected, cancer patients differed from controls in a wide range of inflammatory markers, organ markers, and markers of general nutritional status (data not shown). Especially patients with squamous NSCLC exhibited a distinct pattern in their biochemical profile, suggesting a higher degree of inflammation than in patients with other subtypes.

To assess if the included cases were representative of the total group of patients diagnosed with lung cancer in the study period, patients were compared to non-participating patients. The distribution of non-included and included patients on the various histological diagnoses as well as disease stages did not differ (data not shown).

### PAPP-A2, but Not PAPP-A, Is Elevated in Patients With Lung Cancer and Differ Between Subtypes

**Figure 2:**
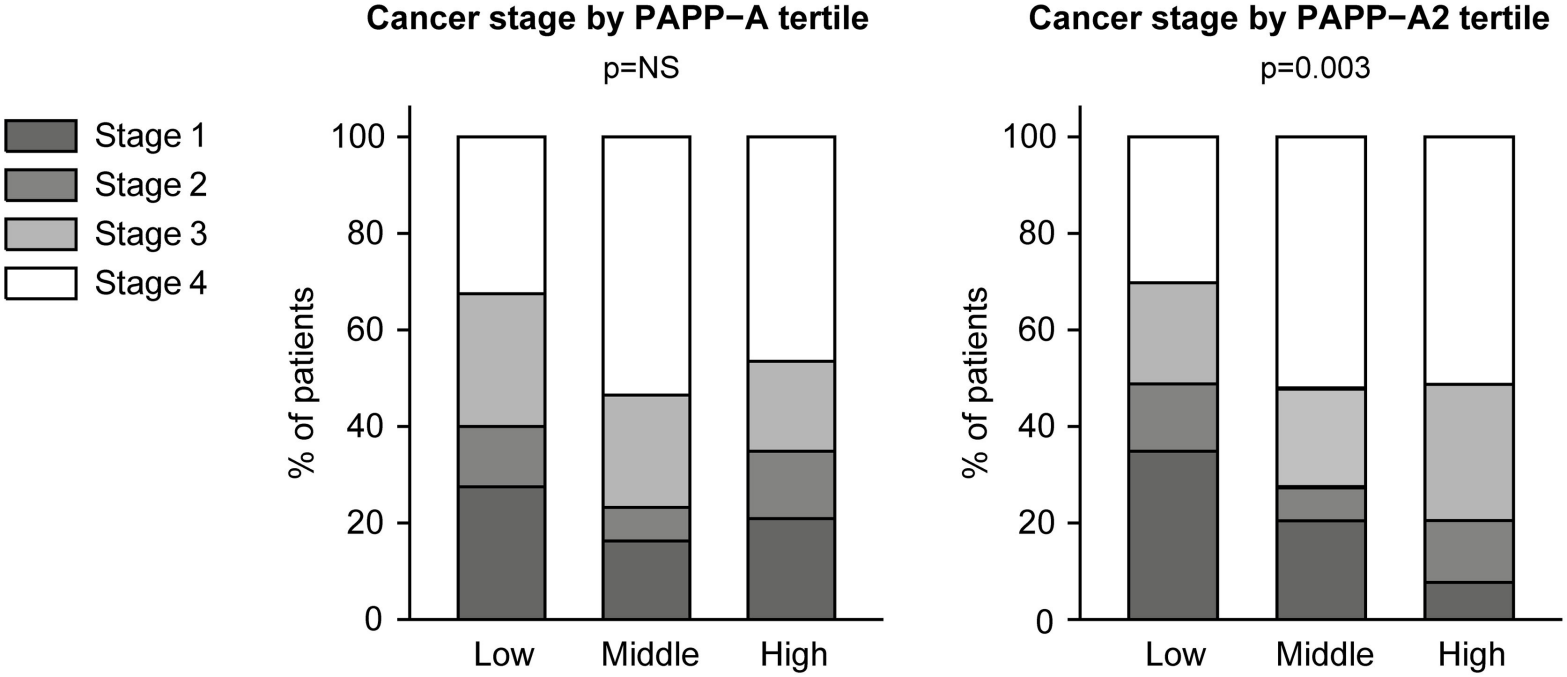
Cancer stage according to PAPP-A and PAPP-A2 tertiles. PAPP-A, pregnancy-associated plasma protein-A.

Serum PAPP-A2 was elevated in patients with lung cancer as compared to control subjects (*p* < 0.001) (Table 1). When comparing the individual cancer subtypes with controls, the highest concentrations were observed in the NS-Sq group (*p* < 0.001). Levels in the NS-x group were also elevated (*p* < 0.05), whereas PAPP-A2 was not significantly higher in the other cancer subtypes. By contrast, PAPP-A levels were similar in cancer and control subjects and did not differ among cancer subtype or stage. PAPP-A2 as a continuous variable was not associated with cancer stage (*p* = 0.123), and thus, patients with early-stage cancer displayed similar concentrations as patients with more advanced-stage cancer. However, when assessing PAPP-A2 tertiles, a higher tertile was significantly associated with higher cancer stage (p_trend_ = 0.003). Cancer stage according to PAPP-A and PAPP-A2 tertiles is shown in Figure 2.

The group of NS-Ad patients was of sufficient size to allow for further analyses of cancer TNM classification. Overall group differences as well as linear trend across TNM category were assessed. However, neither PAPP-A nor PAPP-A2 was associated with tumor size, lymph node involvement or metastatic status.

PAPP-A was positively associated with age (*r* = 0.27, *p* < 0.001) in all subjects, whereas PAPP-A2 was positively associated with age (*r* = 0.40, *p* < 0.001) and negatively associated with BMI (*r* = −0.19, *p* < 0.001). Additionally, in the cancer patients, PAPP-A2 showed correlations with several markers of inflammation, organ status and overall illness, including C-reactive protein (CRP) (*r* = 0.34, *p* < 0.001), erythrocyte sedimentation rate (ESR) (*r* = 0.29, *p* < 0.001), eGFR (*r* = −0.18, *p* < 0.05) and hemoglobin (*r* = −0.30, *p* < 0.001). PAPP-A2 was also positively associated with levels of leukocytes, neutrophils, and monocytes (all *p* < 0.05).

### Immunohistochemistry

**Figure 3:**
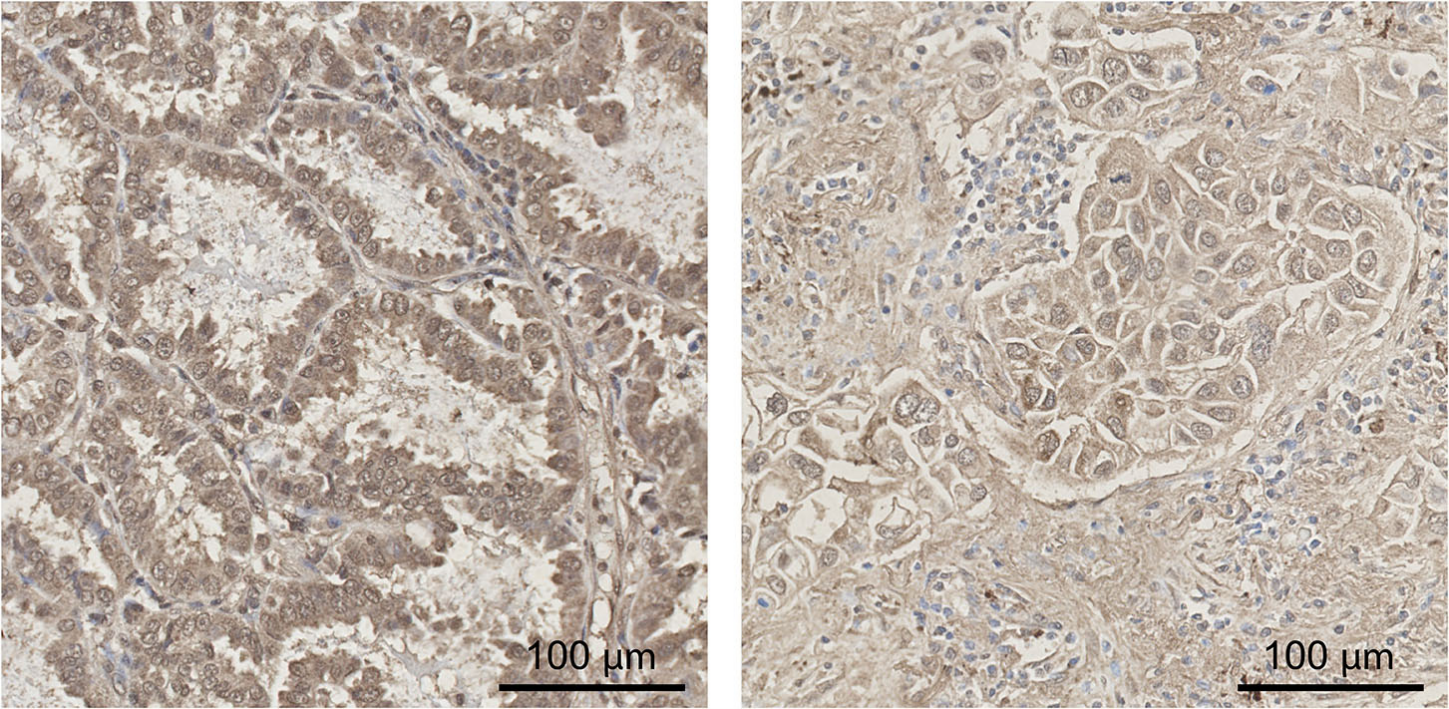
Immunohistochemical staining of PAPP-A2. Expression of PAPP-A2 in lung cancer tissue was determined by immunohistochemical staining. Examples are shown for two patients with non-small cell lung cancer of adenocarcinoma subtype. PAPP-A2 staining was present in malignant cells in recognizable glandular patterns as well as areas densely infiltrated by macrophages. Staining was moderate (left) and weak (right) and occurred in a heterogeneous pattern. Scale bar = 100 μm. PAPP-A2, pregnancy-associated plasma protein-A2.

The expression of PAPP-A and PAPP-A2 was confirmed by IHC of tumors removed during surgery. Tissue originated from one patient with SCLC, two with NS-Ad, and two with NS-Sq. The anti-PAPP-A antibody stained tumor specimens in a vacuole-like or cell membrane accentuated pattern, as expected for a secreted protease, and staining intensity varied across cell types and between patients. Staining for PAPP-A2 was demonstrated in four out of five patients, with no staining in the SCLC tumor sample. PAPP-A2 staining was present in malignant cells as well as areas densely infiltrated by macrophages. Staining was mild to moderate and occurred in a heterogeneous pattern. Examples of the breadth and intensities of PAPP-A2 staining patterns are illustrated in Figure 3.

### Survival Analyses of Cancer Patients

During follow-up, 114 patients (79.2%) died, and median (range) survival of all cancer patients was 377 days (14 days−8 years) (Table 1). Median survival of patients was 550 days in SCLC, 397 days in NS-Ad, 281 days in NS-Sq and 307 days in NS-x. There was no difference in overall mortality between cancer subtypes; 76.9% in SCLC, 78.7% in NS-Ad, 85.2% in NS-Sq and 75.9% in NS-x (*p* = 0.672).

**Figure 4:**
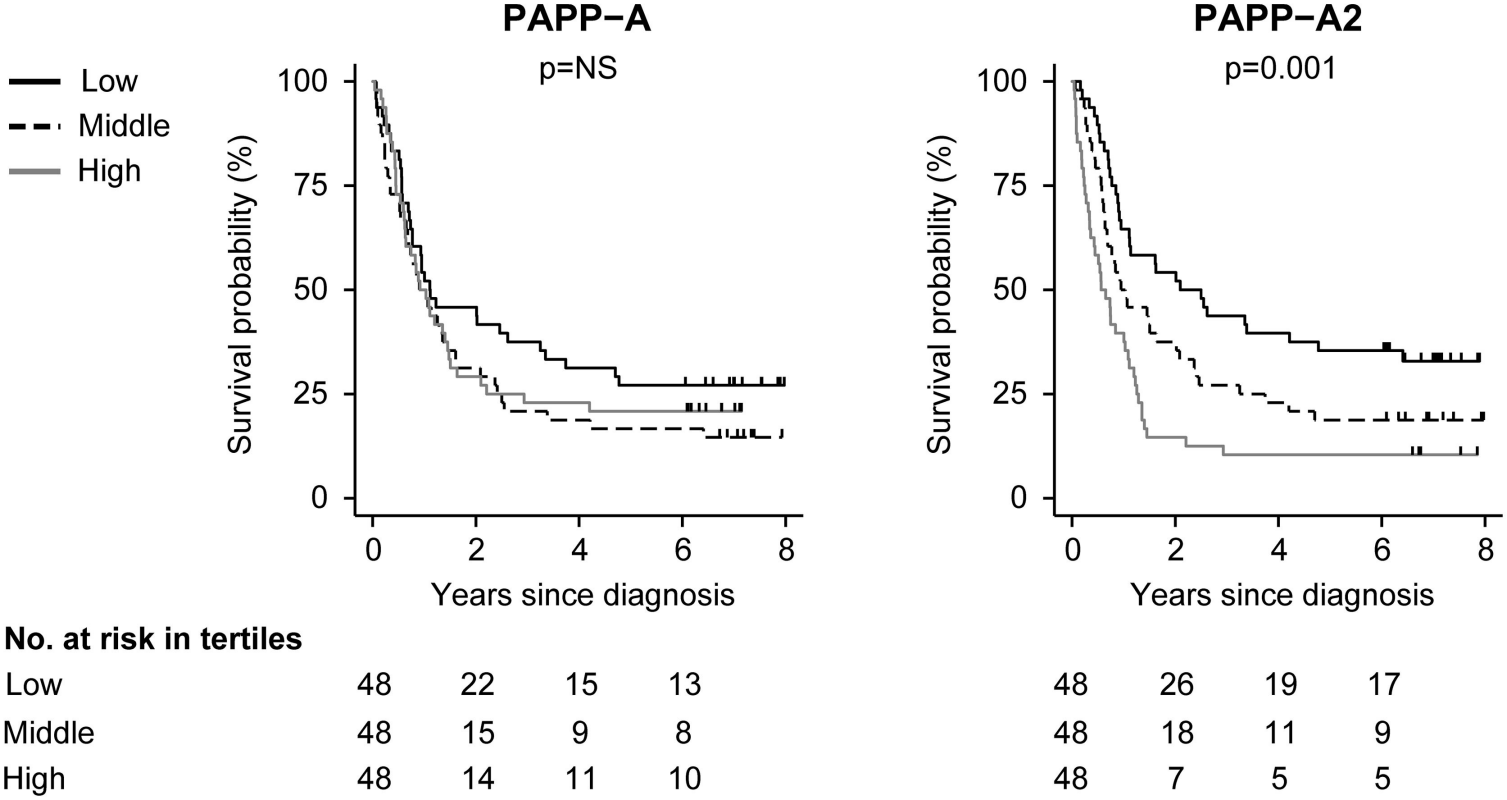
All-cause mortality in patients according to tertiles of PAPP-A and PAPP-A2. Tick marks represent censored events. *P*-values: log-rank test for equality of survival between tertile groups. PAPP-A, pregnancy-associated plasma protein-A.

ROC AUC was 0.55 (0.43; 0.67) for PAPP-A and 0.63 (0.52; 0.73) for PAPP-A2. Log-rank analysis showed similar mortality in the low, middle and high PAPP-A tertiles (*p* = 0.324). However, incidence distributions differed significantly according to PAPP-A2 tertile (*p* < 0.001), and mortality increased with increasing PAPP-A2 tertile (p_trend_ < 0.001). Mortality distribution in the PAPP-A2 tertiles did not differ among the various lung cancer subtypes (*p* = 0.341). Log-rank test and deaths among the various lung cancer subtypes are shown in Table 2. To suggest and illustrate a future potential clinical utility of PAPP-A and PAPP-A2 as biomarkers, Kaplan-Meier survival curves were constructed according to tertiles of PAPP-A and PAPP-A2 (Figure 4). To further investigate the prognostic power of PAPP-A and PAPP-A2, we calculated Harrell's C index, which assesses discrimination ability of survival models. Harrell's C index for PAPP-A was 0.52 (0.46; 58), whereas that for PAPP-A2 was 0.62 (0.57; 0.68).

**Table 2:** Log-rank analyses on all-cause mortality according to PAPP-A or PAPP-A2 tertiles.

**Log-rank**	**Concentration (ng/mL)**	**Total patients (n)**	**SCLC (n)**	**NS-Ad (n)**	**NS-Sq (n)**	**NS-x (n)**	**All-cause mortality (n)**	***p***
**PAPP-A**	0.77 [0.57; 0.86]	48	3	19	8	5	35	0.324
	1.04 [0.98; 1.12]	48	2	24	7	8	41	
	1.54 [1.36; 1.77]	48	5	16	8	9	38	
	p_trend_							0.322
**PAPP-A2**	0.18 [0.13; 0.22]	48	3	20	4	5	32	<0.001
	0.33 [0.28; 0.37]	48	2	19	7	11	39	
	0.69 [0.57; 0.88]	48	5	20	12	6	43	
	p_trend_							<0.001

*Number of events in low, middle and high PAPP-A or PAPP-A2 tertile groups. Median (25th percentile; 75th percentile) concentrations of PAPP-A and PAPP-A2 are shown for each tertile group. In addition, deaths among the various lung cancer subtypes are shown. Values are reported a numbers (n) of patients. P-values, log-rank test for equality of survivor function or test for trend of survivor function across ordered tertile groups (p_trend_)*.

The association between mortality and PAPP-A or PAPP-A2 was investigated using both the continuous variable and tertiles with the low tertile as reference group (Table 3). PAPP-A was not associated with outcome. In contrast, with each 2-fold increase in PAPP-A2, the mortality increased by 30% [HR: 1.30 (1.12; 1.53), *p* = 0.001]. In a categorical model using the first tertile as reference, PAPP-A2 was associated with mortality with a HR of 1.57 (0.98; 2.50), *p* = 0.060, for the second tertile and 2.60 (1.64; 4.14), *p* < 0.001, for the third tertile. In multivariable Cox regressions adjusted for age, sex, and BMI, PAPP-A2 as a continuous variable remained predictive of the endpoint, whereas PAPP-A2 as a categorical variable remained significant when the high tertile was compared to the low tertile.

**Table 3:** Cox regression analyses.

	**Range (ng/mL)**	**Univariable HR (95% CI)**	***p***	**Multivariable HR (95% CI)**	***p***
**ALL-CAUSE MORTALITY**					
**PAPP-A**					
Continuous^a^		1.13 [0.84;1.51]	0.435	1.09 [0.80;1.48]	0.574
Categorical^b^^,^ ^d^					
Low tertile	0.77 [0.57; 0.86]	Reference		Reference	
Middle tertile	1.04 [0.98; 1.12]	1.41 [0.89; 2.21]	0.139	1.32 [0.82; 2.10]	0.251
High tertile	1.54 [1.36; 1.77]	1.26 [0.79; 1.99]	0.328	1.16 [0.729; 1.87]	0.545
**PAPP-A2**					
Continuous^a^		1.30 [1.12; 1.53]	0.001	1.25 [1.05; 1.48]	0.013
Categorical^b^^,^ ^d^					
Low tertile	0.18 [0.13; 0.22]	Reference		Reference	
Middle tertile	0.33 [0.28; 0.37]	1.57 [0.98; 2.50]	0.060	1.51 [0.94; 2.43]	0.086
High tertile	0.69 [0.57; 0.88]	2.60 [1.64;4.14]	<0.001	2.12 [1.26; 3.58]	0.005

*PAPP-A and PAPP-A2 were investigated both in univariable analyses and in multivariable analyses extended by covariables age, sex, and BMI. BMI, body mass index; CI, confidence interval; HR, hazard ratio; PAPP-A, pregnancy-associated plasma protein-A*.

a *Hazard ratio (HR) per doubling of the protein; modeled as log(marker)/log(2). Modeled using Cox proportional hazards regression*.

b *Hazard ratio with the low tertile as reference group. Modeled using Cox proportional hazards regression*.

d *For PAPP-A and PAPP-A2, low, middle and high tertile refers to the lowest, middle and highest tertiles of the protein*.

## Discussion

This prospective study sought to investigate PAPP-A and PAPP-A2 in patients with lung cancer and evaluate potential associations with mortality. PAPP-A2, but not PAPP-A, was elevated in patients with lung cancer, and we demonstrated a prognostic significance of PAPP-A2. The present study is the first exploration of the potential clinical significance of PAPP-A2 in this disease. However, the novel association between PAPP-A2 and lung cancer warrants further validation in external cohorts as well as functional studies to establish a causal relationship.

Early detection and treatment of lung cancer are urgent global healthcare priorities and pose a formidable challenge. Unfortunately, early symptoms, if present, are indistinct and non-specific, and the majority of patients appear with advanced disease. Thus, novel ways to identify patients and treatment options are crucial. IGF signaling clearly plays a pivotal role in the progressive transformation of normal cells into malignant derivatives and has been shown to regulate most steps of tumor progression, including sustained cell proliferation, clonal expansion, angiogenesis, migration, invasion, and colonization of secondary sites and resistance to certain anti-cancer therapies (2). PAPP-A has emerged as an oncogene, and burgeoning evidence indicates that PAPP-A is implicated in tumor formation through the amplification of IGF actions. PAPP-A is expressed by a wide range of cells of malignant origin (31, 32), being transiently increased in some cancers and constitutively expressed by others (8, 20, 33). In murine models, PAPP-A deficiency results in a delayed occurrence of age-related fatal cancers and sporadic tumors (34, 35). In 2009, Bulut et al. found increased PAPP-A levels in serum from patients with lung cancer (19). However, we were unable to confirm this finding in the present study. Furthermore, PAPP-A was not associated with mortality and does not appear to possess potential as a seromarker in a heterogeneous cohort of lung cancer patients.

In view of the roles of PAPP-A in neoplasia, we also examined its homolog, PAPP-A2. Proteolytic activity against IGFBP-5 has been reported in various fluids and cells from several sources (4, 36–39). In mice, genetic deletion of PAPP-A2 results in normal size at birth, but there is postnatal growth retardation and bone abnormalities (23). Recently, a novel loss-of-function mutation in the human *PAPPA2* gene was discovered, resulting in a syndrome of growth retardation with elevated concentrations of IGFs, but a decreased bioactivity due to a concomitant increase in serum IGFBP-3 and−5 (14). These are the first human cases of reduced IGF-I bioavailability caused by defects in IGFBP regulation, demonstrating that PAPP-A2 has relevant consequences in human growth. Furthermore, the study confirmed the absence of functional redundancy between PAPP-A and PAPP-A2. In regard to cancer, few studies mention PAPP-A2. In 2013, a whole-exome sequencing study of lung adenocarcinoma patients identified *PAPPA2* gene mutations that were associated with prolonged survival times (40). In 2017, the present authors investigated PAPP-A, PAPP-A2 and IGF activity in pleural fluid collected at baseline from a limited number of patients with lung cancer (*n* = 24) (18). The study showed that the distribution of IGF system proteins in pleural effusions was substantially different from that of the circulating IGF system. As compared to serum, pleura contained 47-fold higher concentrations of PAPP-A and 3.3-fold higher concentrations of PAPP-A2. Although total IGF-I levels in pleura and serum were comparable, levels of free IGF-I and the ability of pleural fluid to activate the IGF-IR *in vitro* was more than 3-fold higher. These findings support that not only PAPP-A, but also PAPP-A2, modulate the IGF signaling cascade in cancers, and furthermore, indicate that the local activity of the IGF system in extravascular fluids differs substantially from that of the circulating IGF system. Finally, our previous findings support the hypothesis that PAPP-A and PAPP-A2 regulate IGF activity without affecting total IGF-I levels.

In the present study, PAPP-A2 levels were higher in patients with lung cancer than controls. Furthermore, the presence of PAPP-A2 in cancerous tissue was demonstrated by IHC and PAPP-A2 possessed prognostic ability. Further studies to test the hypothesis could lead to the establishment of PAPP-A2 as a diagnostic and prognostic biomarker. In addition, it is reasonable to assume that increased serum levels of PAPP-A2 in lung cancer patients may correlate with augmented IGF signaling in tumor cells, and thus, PAPP-A2 may also possess potential as a biomarker for IGF-I targeted therapy. Interestingly, higher PAPP-A2 levels were not unambiguously associated with advanced stages of tumor development, suggesting that the increase in PAPP-A2 may be present even at early stages. Only when assessing PAPP-A2 tertiles, an association with tumor stage was seen. By scrutinizing PAPP-A2 levels, it was clear that some patients exhibited significantly higher PAPP-A2 levels than others, and that levels differed between tumor subtypes. We speculate that the secretion of PAPP-A2 may be elevated in some tumors, whereas others do not express PAPP-A2 at a higher level than non-cancerous lung tissue. This notion is further supported by the fact that IHC staining of PAPP-A2 was only demonstrated in four out of five patients, and that staining intensity was heterogeneous and varied considerably across cell types and between patients. Of interest, staining was lacking in the one patient with SCLC. The clinically most important division is between SCLC and NSCLC, and the lack of PAPP-A2 staining supports the concept of different cancerous mechanisms in SCLC and NSCLC. IHC analysis was, however, only performed in five patients, and more patients are needed to further investigate these speculations. Nevertheless, such tumor heterogeneity has previously been shown to apply to PAPP-A. Studies have demonstrated that some malignant cells show higher proclivity toward expressing PAPP-A than others. In women with breast cancer, overexpression of PAPP-A was observed in 79% (8), and various subtypes revealed extensive IHC staining of PAPP-A in 45 of 46 specimens (41). The *PAPPA* gene is located in a chromosomal region associated with high frequency of loss of heterozygosity in ovarian tumors (42). However, in a study of lung cancer cell lines, PAPP-A was only secreted from two out of seven (21). In mice with patient ovarian tumor grafts, PAPP-A inhibition with a neutralizing antibody showed beneficial effects only in tumors expressing moderate-to-high levels of PAPP-A (7). These findings imply that PAPP-A secretion from cancer cells contributes to growth in a tumor-specific manner, and the same may very well be the case for PAPP-A2. The growth of some malignant tumors may not be under the influence of PAPP-A2 or may only be affected by PAPP-A2 secreted from non-malignant neighboring cells or by PAPP-A2 of endocrine origin, which is present in the tumor environment. Finally, the activity of PAPP-A2 in the promotion of tumor growth is tied to the capability of cells to not only secrete functional PAPP-A2, but also express IGF-IRs. Dysregulation of signaling pathways in differentiating cells can dictate the emergence of neoplastic cells, but tumors have a diverse genetic makeup that renders them reliant on different signaling pathways for growth. Indeed, the IGF signaling pathway is only one of many, and the large number of driving forces behind different cancer subtypes is poorly understood. Collectively, inter-tumoral variability makes interpretations of the pathophysiology of PAPP-A2 difficult. Most current therapies treat cancer as a homogenous disease and customizing anti-cancer therapies to target specific neoplasms presents an ongoing challenge in the field of cancer therapy.

The increase in PAPP-A and PAPP-A2 in tumors and tumor microenvironments may also be reflective of other pathological processes. PAPP-A is often associated with inflammatory states, and levels are up-regulated by several pro-inflammatory cytokines, with IL-1β and tumor necrosis factor (TNF)-α being invariably potent promoters (43, 44). Aberrant immune responses are involved in cancer patients before clinical confirmation of disease, and chronic inflammation predisposes to and is involved in the onset of tumorigenesis (45). Furthermore, an inflammatory microenvironment has been suggested to induce proliferation of neoplastic cells (46). The mechanism as to how inflammatory signals exacerbate malignant development is poorly understood. In a cancer setting, an inflammatory milieu may potentiate PAPP-A and perhaps PAPP-A2 expression, further encouraging tumor growth. However, in this study, PAPP-A did not appear associated with markers of inflammation, and levels were not higher in NS-Sq patients, although this group displayed a higher degree of inflammation than patients with other subtypes. On the contrary, PAPP-A2 showed correlations with several markers of inflammation, and the highest levels of PAPP-A2 were found in the NS-Sq group. Thus, high PAPP-A2 levels may reflect an inflammatory state as well as cancer disease.

The IGF system and its protease system is an exciting area of research that could spur progress in cancer diagnostics and treatment, and it is conceivable that PAPP-A2 neutralizing antibodies would show beneficial effect in cancer therapy. However, most important are studies into the specific driving forces behind different subtypes and intra-tumor heterogeneity, which will facilitate a better understanding of the nature of cancer and provide insight into the development of more effective and personalized cancer therapies.

Some strengths and limitations of our study should be acknowledged to aid in data interpretation. A primary strength is the Danish nationwide health registers that offer ideal opportunities for epidemiological research. Registration of cancer cases in Denmark is mandatory and provides complete follow-up, the ascertainment of lung cancer is near complete and the free public healthcare system essentially eliminates private hospital treatment. However, this is a single center, prospective cohort study design, and although prospective studies usually have fewer potential sources of bias and confounding than retrospective studies, our results must be evaluated in light of that. Samples were collected at baseline with no measurement beyond, and thus, we were unable to evaluate dynamic changes in protein levels over time. Furthermore, the small number of cancer patients in some of the histological subgroups did not encourage profound subdivisions. The control group comprised non-cancer patients but cannot be considered healthy participants. All were referred due to suspected lung cancer but held a variety of other diagnoses. Finally, to ensure that PAPP-A and PAPP-A2 levels were analyzed in the context of lung cancer and not another undiscovered cancer disease, we excluded control patients who had an incident cancer diagnosis at another site during the first 2 years of follow-up. Eventually, our studies must be validated in a second independent cohort, and functional and mechanistic studies must be performed to establish causal relationships.

## Conclusion

In lung cancer patients, PAPP-A2 emerged as a predictor of mortality, levels were increased as compared to controls and PAPP-A2 expression was documented in malignant tissues. PAPP-A2 may induce augmented IGF signaling in tumor cells, and further studies to test this hypothesis could lead to the establishment of PAPP-A2 as a prognostic marker or a biomarker for IGF-I targeted therapy. To confirm this association, our studies must be validated in new and external cohorts in the future.

## Data Availability Statement

The raw data supporting the conclusions of this article will be made available by the authors, without undue reservation.

## Ethics Statement

The studies involving human participants were reviewed and approved by Central Denmark Region Committees on Biomedical Research Ethics (IRB 0005129). The patients/participants provided their written informed consent to participate in this study.

## Author Contributions

RH, UE, and JF conceived and designed the study. UE, TR, and BF were responsible for patient recruitment and sample collection. RH, UE, TS, JG, and CO performed the experiments and acquired the data. Data interpretation and statistical analyses were performed by RH and UE. RH drafted the manuscript, which was revised and approved by all authors. RH had full access to all data in the study and takes responsibility for the integrity of the data and the accuracy of the data analysis. All authors contributed to the article and approved the submitted version.

## Conflict of Interest

The authors declare that the research was conducted in the absence of any commercial or financial relationships that could be construed as a potential conflict of interest.
